# Identifying faulty brain circuits

**DOI:** 10.7554/eLife.26942

**Published:** 2017-04-25

**Authors:** Jesse E Hanson

**Affiliations:** Department of Neuroscience, Genentech, Inc., San Francisco, United Stateshanson.jesse@gene.com

**Keywords:** immediate early gene, inhibitory neuron, dementia, Alzheimer's disease, Human, Mouse

## Abstract

A protein called NPTX2 may be a useful marker of neural circuit defects in patients with Alzheimer’s disease.

**Related research article** Xiao M-F, Xu D, Craig MT, Pelkey KA, Chen C-C, Shi Y, Zhang J, Resnick. S, Pleknikova O, Salmon D, Brewer J, Edland S, Wegiel J, Tycko B, Savonenko A, Reeves RH, Troncosco JC, McBain CJ, Galasko D, Worley PF. 2017. NPTX2 and cognitive dysfunction in Alzheimer’s Disease. *eLife* **6**:e23798. doi: 10.7554/eLife.23798

Individuals with Alzheimer’s disease experience memory loss, and find it difficult to make decisions and learn new things. These cognitive impairments begin in the early stages of the disease and are the result of changes to neurons and the connections between them. Accumulating evidence suggests that these changes involve inhibitory interneurons, which normally shape and restrain the activity of circuits of neurons, becoming less active (Palop and Mucke, 2016). However, it is not fully understood what causes the loss of interneuron activity in Alzheimer’s disease. Furthermore, it is hard to identify if interneurons have been affected in specific patients because there are currently no known molecular biomarkers of interneuron activity.

Now, in eLife, Paul Worley from the Johns Hopkins University School of Medicine and co-workers – including Mei-Fang Xiao and Desheng Xu as joint first authors – report that the loss of a protein called NPTX2 disrupts interneuron-mediated circuit activity in Alzheimer’s disease (Xiao et al., 2017). They also put forward NPTX2 as a biomarker that could be used to measure defects in neural circuits seen in patients with the disease.

NPTX2 (also known as NARP) is released from excitatory neurons and binds to receptors on interneurons that are characterized by the expression of another protein called parvalbumin (Chang et al., 2010). These receptors, known as AMPA receptors, activate the neurons; thus NPTX2 is important in maintaining the normal activity of the parvalbumin interneurons, which prevents certain neural circuits from becoming too active (Gu et al., 2013). Xiao et al. found that patients with Alzheimer’s disease have less NPTX2 in their brains compared to healthy individuals (Figure 1). The brains of these patients also have lower levels of a subunit of the AMPA receptors called GluA4 (which is preferentially expressed in parvalbumin interneurons).

**Figure 1.**
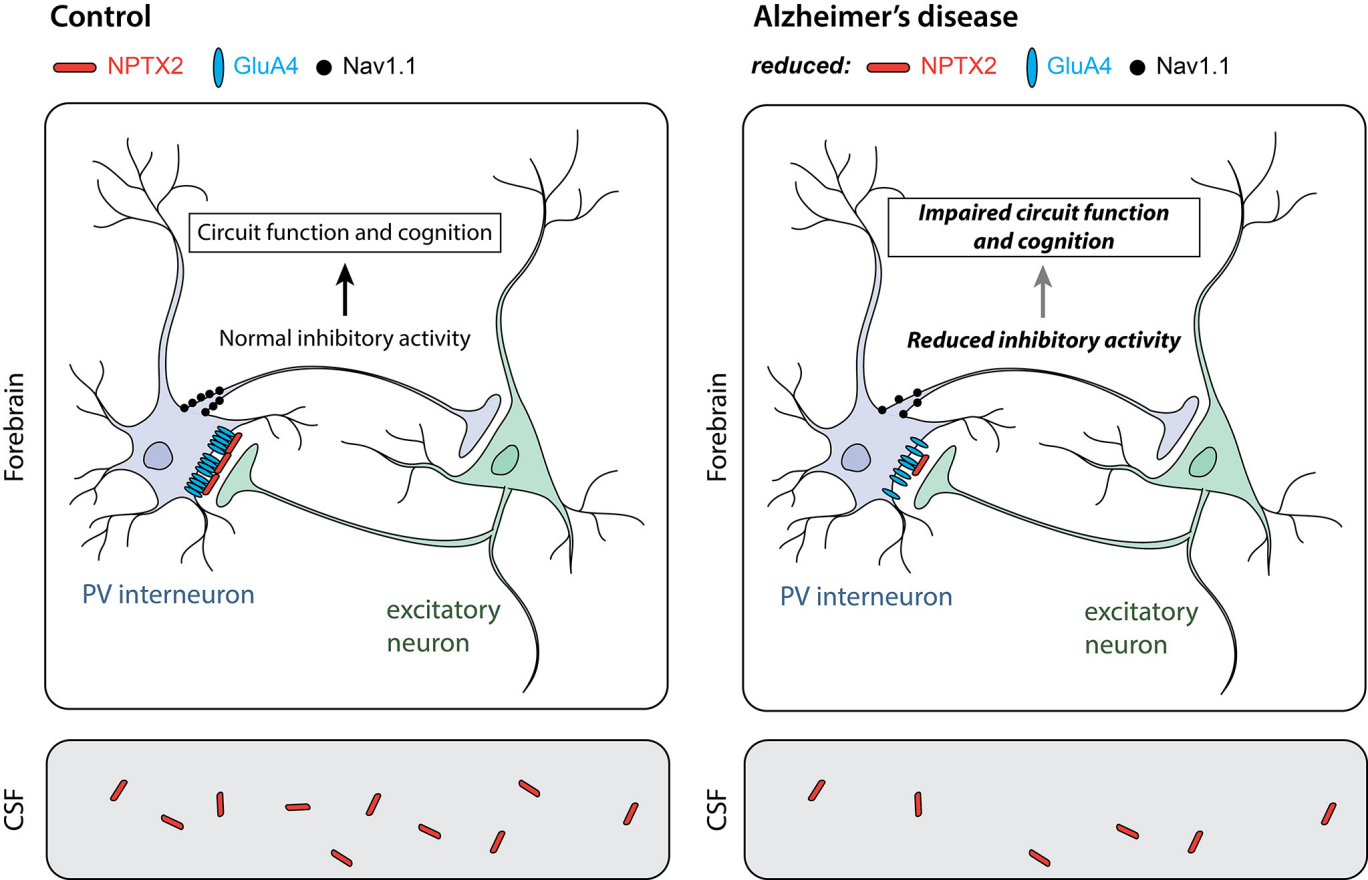
Parvalbumin interneurons in Alzheimer’s disease. Parvalbumin interneurons (PV interneuron) connect to excitatory neurons in the forebrain (top). In healthy individuals (control; left), the NPTX2 protein (red) arranges GluA4-containing AMPA receptors (blue) in clusters at these connections. As a result, electrical signals from the excitatory neurons strongly activate the interneurons. A sodium channel called Nav1.1 (black) in the interneurons helps to generate electrical signals that inhibit other neurons in the circuit. In the brains of individuals with Alzheimer’s disease (right), the levels of NPTX2, GluA4 and Nav1.1 are all lower than in healthy individuals; this leads to less inhibitory interneuron activity. Other neurons in the circuit thus become more active than they should be, resulting in defects in circuit function and cognitive impairments. NPTX2 can also be detected in the cerebrospinal fluid (CSF; bottom). Individuals with Alzheimer’s disease (right) have less NPTX2 in their CSF compared to healthy individuals (control; left). NPTX2 levels in CSF correlate with cognitive impairments.

IMAGE CREDIT: JE Hanson, A Bruce.

A fundamental feature of Alzheimer’s disease is the accumulation of a peptide called amyloid beta in the brain in a process called amyloidosis. To explore how NPTX2 might be interacting with amyloidosis, Xiao et al. turned to mouse models. They found that mice with amyloidosis and deletion of the NPTX2 gene had lower levels of GluA4 and more defects in circuit activity than mice with just amyloidosis alone. Xiao et al. also observed that humans with amyloidosis, but not Alzheimer’s disease, have normal levels of NPTX2 in their brain. Together these results support the idea that amyloidosis does not immediately cause a decrease in NPTX2 expression, but rather that a drop in NPTX2 could be a “second hit” that collaborates with amyloidosis to lead to the defects in neural circuits and cognition seen in Alzheimer’s disease.

Other observations also support the model in which parvalbumin interneurons being less active causes defects in neural circuits in Alzheimer’s disease. For example, patients with Alzheimer’s disease and mouse models of the disease also have reduced levels of a sodium channel called Nav1.1, which is critical for parvalbumin interneuron activity (Verret et al., 2012). Restoring Nav1.1 to normal levels improves circuit activity and reduces cognitive impairments in the mouse models. A recent study shows that enhancing parvalbumin interneuron activity in a mouse model of Alzheimer’s disease can decrease the accumulation of amyloid peptides in the brain (Iaccarino et al., 2016). These studies suggest that reduced activity of parvalbumin interneurons may contribute to the symptoms and pathology of Alzheimer’s disease.

Xiao et al. next assessed NPTX2 in human cerebrospinal fluid and found reduced levels in Alzheimer’s disease patients (Figure 1). They found that NPTX2 levels performed as well as current biomarkers of the disease in terms of sensitivity and specificity in distinguishing patients from healthy controls. Xiao et al. also found that NPTX2 levels in cerebrospinal fluid correlated with cognitive performance in the patients. This is consistent with another recent study showing that Alzheimer’s disease patients with higher initial levels of NPTX2 in their cerebrospinal fluid experience less memory decline over a two-year period (Swanson and Willette, 2016).

The ability to detect reduced NPTX2 in cerebrospinal fluid is relevant because targeting parvalbumin interneurons in particular, and circuit defects in general, is emerging as a potential way to treat cognitive impairments in patients with Alzheimer’s disease (Palop and Mucke, 2016). For example, an ongoing clinical effort is testing an anticonvulsant drug called levetiracetam as a treatment for cognitive impairment (NCT02002819; Bakker et al., 2012) based on observations in patients (Yassa et al., 2010) and mouse models (Sanchez et al., 2012). Using NPTX2 as a biomarker might help such clinical efforts to identify patients who may have neural circuit defects.

More work is needed to understand the root causes of the decreases in NPTX2 and GluA4 levels in Alzheimer’s disease. Clinical experiments examining brain activity in Alzheimer’s patients would help to confirm that reduced NPTX2 levels are actually associated with neural circuit defects in human patients (and not just mouse models).
